# Consistent Brain Entropy Changes During Rumination Across Three Magnetic Resonance Imaging Scanners

**DOI:** 10.1155/np/7238736

**Published:** 2026-06-01

**Authors:** Jue Lu, Donghui Song, Da Chang, Xiao Zhang, Xiaoye Ma, Ze Wang

**Affiliations:** ^1^ School of Mathematics, Physics and Information Science, Shaoxing University, Shaoxing, 312000, China, usx.edu.cn; ^2^ Department of Diagnostic Radiology and Nuclear Medicine, University of Maryland School of Medicine, Baltimore, 21201, Maryland, USA, umd.edu; ^3^ College of Education Science, Hengyang Normal University, Hengyang, 421008, China, hynu.edu.cn; ^4^ Peking University Sixth Hospital, Beijing, 100191, China; ^5^ Peking University Institute of Mental Health, Beijing, 100191, China, pku.edu.cn; ^6^ Shanghai Mental Health Center, Shanghai Jiaotong University, Shanghai, 200030, China, sjtu.edu.cn

**Keywords:** brain entropy (BEN), fMRI, posterior cingulate cortex, precuneus, rumination, visual cortex

## Abstract

Rumination, characterized by repetitive and self‐referential thinking, is closely linked to depression and a broad range of psychiatric disorders. However, previous research has predominantly relied on task activation or network‐level approaches, leaving critical gaps in our understanding of local brain activity dynamics during ruminative states. To address this, we employed brain entropy (BEN), a novel functional magnetic resonance imaging (fMRI)–based neuroimaging measure, to quantify the temporal irregularity and complexity of local brain activity during rumination. We analyzed a publicly available dataset comprising 41 healthy adults who completed identical fMRI tasks across three MRI scanners. Each scanning session included four conditions: resting state, sad memory, rumination, and distraction. Voxel‐wise BEN analyses were conducted to examine condition‐related differences, with brain regions showing consistent patterns across all three scanners at *p* < 0.05 considered statistically significant. Our findings revealed distinct neural signatures associated with different cognitive states. Compared to sad memory, rumination was associated with decreased BEN in the visual cortex (VC), whereas distraction was associated with decreased BEN in the posterior cingulate cortex/precuneus (PCC/PCu). Furthermore, rumination exhibited significantly increased BEN in the PCC/PCu relative to distraction. These results suggest that rumination is characterized by heightened temporal irregularity in regions supporting internal self‐referential processing, accompanied by reduced engagement with external environmental information. The present study demonstrates the utility of BEN in elucidating the neural mechanisms of rumination, providing novel insights into how cognitive states are reflected in local brain activity patterns. These findings carry implications for both theoretical frameworks of ruminative cognition and the development of neuroimaging biomarkers for clinical applications in depression and related disorders.

## 1. Introduction

Rumination is defined as recurrent and repetitive thinking on symptoms, feelings, problems, upsetting events, and negative aspects of the self, typically with a focus on their causes, circumstances, meanings, and implications [1]. Rumination is closely related to depression and considered a common mechanism relating depressive risk factors to depression [2, 3]. Importantly, rumination is not only related to depression but is also involved in the development and maintenance of a broad range of disorders, including posttraumatic stress disorder (PTSD), anxiety disorders, insomnia, eating disorders, somatic symptom disorder, and substance use disorders, which is increasingly recognized as a transdiagnostic phenomenon [4–7]. A recent review [8] demonstrates that rumination has multiple negative consequences: (1) It exacerbates psychopathology by magnifying and prolonging negative mood states, interfering with problem‐solving and instrumental behavior and reducing sensitivity to changing contingencies; (2) it acts as a transdiagnostic mental health vulnerability impacting anxiety, depression, psychosis, insomnia, and impulsive behaviors; (3) it interferes with therapy and limits the efficacy of psychological interventions; and (4) it exacerbates and maintains physiological stress responses. Understanding the neural substrates underlying rumination can enhance our understanding of a range of psychopathologies and their treatments beyond depression [9]. Noninvasive neuroimaging technologies, such as task‐based functional magnetic resonance imaging (fMRI), have enabled the systematic investigation of these neural substrates. For instance, studies have demonstrated enhanced activation within the default mode network (DMN) during ruminative states across both healthy and clinical populations, with individuals with depression showing even greater activation levels [10, 11]. This association between rumination and the DMN has been substantiated by recent meta‐analytic evidence, which confirms that ruminative cognition is predominantly mediated by DMN [12]. Building upon these activation‐based findings, connectivity analyses have revealed a more nuanced picture of DMN involvement in rumination. Specifically, Chen et al. [13] demonstrated that stable within‐DMN functional connectivity (FC) was significantly attenuated during rumination relative to distraction conditions [13]. While these investigations have substantially advanced our understanding of the neural mechanisms underlying rumination, they have predominantly relied on conventional task activation paradigms and static network connectivity analyses [12–14]. This methodological focus has left a critical gap in our knowledge regarding the temporal dynamic characteristics of local brain activity during ruminative states. Addressing this limitation requires novel analytical approaches that can capture the moment‐to‐moment fluctuations in neural activity that may characterize ruminative cognition. The present study aimed to investigate the neural substrates of rumination by examining local brain dynamics through fMRI‐derived brain entropy (BEN) analysis [15], utilizing publicly accessible data from the RMP Rumination fMRI Dataset (http://rfmri.org/RuminationfMRIData) [13]. This analytical approach offers novel insights into the temporal complexity and irregularity of neural activity patterns during ruminative states.

Entropy quantifies the irregularity, disorder, and complexity of dynamic systems [16–19]. As a self‐organizing, adaptive functional system, the brain maintains entropy within an optimal range, characterized by relatively low overall entropy with substantial regional heterogeneity [15, 20, 21]. The fMRI‐based BEN mapping provides a versatile framework for quantifying regional entropy patterns and their associations with neurobiological indices [15, 22]. Critically, BEN captures unique dimensions of neural activity that are not accessible through conventional neuroimaging measures, including cerebral perfusion and the amplitude of low‐frequency fluctuations in fMRI [23], thereby establishing its distinctive utility for neural function assessment. This methodological advantage positions BEN as a complementary biomarker that reveals previously undetectable aspects of brain dynamics.

Extensive research has suggested that BEN as a sensitive and reliable biomarker across diverse domains of brain function [24–31]. Previous research has demonstrated significant associations between fMRI‐derived BEN and neurocognitive performance [24, 26] as well as task activation [25, 27]. Furthermore, BEN exhibits remarkable sensitivity to neurochemical signaling [30, 31] and various neuromodulation protocols [29, 32–34]. Disease‐related BEN alterations have also been documented across a comprehensive spectrum of psychiatric and neurological conditions [35–47]. In depression, we have identified several hyperentropy patterns. Symptom severity correlates positively with BEN elevation in the subgenual anterior cingulate cortex and medial orbitofrontal cortex (sgACC/MOFC), with pharmacological treatment normalizing these alterations [38]. Similarly, individuals with mild‐to‐moderate depression exhibit elevated BEN in the prefrontal and limbic networks, which decreases following nonpharmacological interventions [46], and a recent investigation demonstrated elevated BEN in the right precuneus and angular gyrus among depression patients, with significant therapeutic reductions observed following electroconvulsive therapy [43]. Moreover, high‐frequency repetitive transcranial magnetic stimulation (rTMS) targeting the left dorsolateral prefrontal cortex (DLPFC), an established therapeutic protocol for depression [48, 49], reduces BEN in the sgACC/MOFC [32]. In contrast, low‐frequency rTMS increased BEN in the same brain regions [29].

The present investigation leveraged the RMP Rumination fMRI Dataset to examine BEN patterns during rumination. This publicly available dataset provides a methodologically rigorous foundation, having employed identical experimental protocols across three distinct MR scanner platforms (GE and Siemens), thereby enabling robust assessment of finding reproducibility, a critical consideration for establishing reliable neuroimaging biomarkers. We hypothesized that rumination would elicit an increased BEN within the DMN. This prediction integrates convergent evidence from the same dataset demonstrating robust within‐DMN FC reductions during rumination [13] and consistent observations of elevated BEN in depression [37, 38, 43, 46].

## 2. Methods

### 2.1. Dataset

All data were obtained from the publicly available RMP Rumination fMRI Dataset (http://rfmri.org/RuminationfMRIData). The dataset comprises 41 healthy young adults (22 females; mean age = 22.7 ± 4.1 years), with each participant undergoing identical scanning procedures across three different MRI scanner platforms located at the Institute of Psychology, Chinese Academy of Sciences (IPCAS) and Peking University (PKU). All participants provided written informed consent, and the experimental procedures received approval from the Institutional Review Board of the IPCAS, as reported in the original publication [13].

#### 2.1.1. Experimental Design

Each participant underwent scanning using all three MRI systems, with scanner order counterbalanced across participants. Identical fMRI task protocols were administered across all scanner sessions. To minimize habituation effects and assess test–retest reliability over extended intervals, sessions were separated by 22.0 ± 14.6 days between different scanners. Each scanning session comprised four sequential fMRI acquisitions: resting state, sad memory, rumination, and distraction. The resting‐state scan was acquired first, during which participants remained motionless for 8 min with eyes closed and no specific instructions. The sad memory immediately followed the resting‐state acquisition, succeeded by either the rumination or distraction tasks (order counterbalanced across participants). All three mental state conditions (sad memory, rumination, and distraction) consisted of four sequentially presented stimuli.

The sad memory condition involved recalling four distinct negative autobiographical events. Participants received the following task instructions: rumination was defined as “passive and repetitive thinking about negative events and their possible consequences,” while distraction was described as “imagining scenes or images unrelated to yourself” [10, 13, 50]. Each stimulus was presented for 2 min before transitioning to the next stimulus without interstimulus intervals, creating continuous 8‐min mental state for each condition.

#### 2.1.2. MRI Data Acquisition

Images were acquired using three different scanners: a 3 tesla GE MR750 scanner at the Magnetic Resonance Imaging Research Center, IPCAS (IPCASGE); a 3 tesla GE MR750 at PKU (PKUGE); and a 3 tesla Siemens Prisma scanner at PKU (PKUSIEMENS). All scanners were utilized with an 8‐channel coil. Scan parameters followed the recommended standardized sequence of the Association of Brain Imaging (www.abimaging.org), which was developed to harmonize site effects across different scanner models.

All participants underwent a 3D T1‐weighted scan prior to functional image acquisition with the following parameters: 192 sagittal slices, TR = 6.7 ms, TE = 2.90 ms, slice thickness/gap = 1/0 mm, in‐plane resolution = 256 × 256, inversion time (TI) = 450 ms, FOV = 256 × 256 mm, flip angle (FA) = 7°, average = 1 from IPCASGE and PKUGE; 192 sagittal slices, TR = 2530 ms, TE = 2.98 ms, slice thickness/gap = 1/0 mm, in‐plane resolution = 256 × 224, TI = 1100 ms, FOV = 256 × 224 mm, FA = 7°, and average = 1 from PKUSIEMENS. Then, fMRI images were acquired during the resting state and three mental states (sad memory, rumination, and distraction) using the following parameters: 33 axial slices, TR = 2000 ms, TE = 30 ms, FA = 90°, thickness/gap = 3.5/0.6 mm, FOV = 220 × 220 mm, matrix = 64 × 64 from IPCASGE and PKUGE; 62 axial slices, TR = 2000 ms, TE = 30 ms, FA = 90°, thickness = 2 mm, multiband factor = 2, and FOV = 224 × 224 mm from PKUSIEMENS.

More detailed experimental design and data acquisition information can be found in [13] and the dataset webpage (http://rfmri.org/RuminationfMRIData).

### 2.2. MRI Preprocessing and BEN Mapping

#### 2.2.1. MRI Preprocessing

Preprocessing for acquired MR images was performed using statistical parametric mapping (SPM12, Wellcome Trust Centre for Neuroimaging, London, UK, http://www.fil.ion.ucl.ac.uk/spm/software/spm12/) [51]. The preprocessing was performed using the following steps: (1) The first four volumes were discarded to allow the signal to reach steady state; (2) slice timing correction was applied to the remaining images to account for temporal differences in slice acquisition; (3) realignments on functional images were performed using a six‐parameter (rigid body) linear transformation to correct head motion; (4) structural images were segmented into gray matter (GM), white matter (WM), and cerebrospinal fluid (CSF). Functional images were spatially coregistered with structural images, and WM and CSF segmentation maps were back‐registered into the functional image space and resampled to the same resolution for extracting mean WM and CSF signals; (5) temporal nuisance correction was performed by regressing out six head motion parameters, WM signal, and CSF signal. Global signal regression was not performed; (6) temporal bandpass filtering (0.01–0.1 Hz) was performed; and (7) the functional images were smoothed with an isotropic Gaussian kernel with a full‐width‐at‐half‐maximum (FWHM) of 6 mm.

#### 2.2.2. BEN Mapping

BEN mapping was performed using BENtbx (https://cfn.upenn.edu/zewang/software.html) [15]. BEN maps were calculated from the preprocessed functional images at each voxel using sample entropy [52]. BEN mapping parameters were selected following established protocols from previous publications [15, 24, 25], with the window length = 3 and the cutoff threshold = 0.6. The BEN maps were normalized to the Montreal Neurological Institute (MNI) standard space and resampled to 3 × 3 × 3 mm^3^ resolution and then smoothed with an isotropic Gaussian kernel (FWHM = 10 mm). More comprehensive details regarding the BEN calculation methodology can be found in the original BENtbx publication [15].

### 2.3. Statistical Analysis

We first estimated the differences between task‐state and resting‐state BEN using paired *t*‐tests with an initial threshold of *p* < 0.05. Regions showing consistent overlap across all three scanners were considered to exhibit significant differences between task‐state and resting‐state BEN. Subsequent analyses were restricted to these brain regions demonstrating significant task‐related BEN changes. The following contrasts were evaluated using paired *t*‐tests for each scanner: rumination vs. sad memory, distraction vs. sad memory, and rumination vs. distraction. An initial statistical threshold of *p* < 0.05 was applied, and brain regions consistently showing significant differences across all three scanners were identified for further analysis. For regions exhibiting significant between‐condition differences, mean BEN values were extracted for each participant. Individual‐level variability was visualized through plotting and statistically evaluated using paired *t*‐tests to assess the consistency of group‐level findings at the individual participant level. All voxel‐wise statistical analyses were conducted using custom Python scripts based on Nilearn (https://nilearn.github.io/stable/index.html).

## 3. Results

**Figure 1:**
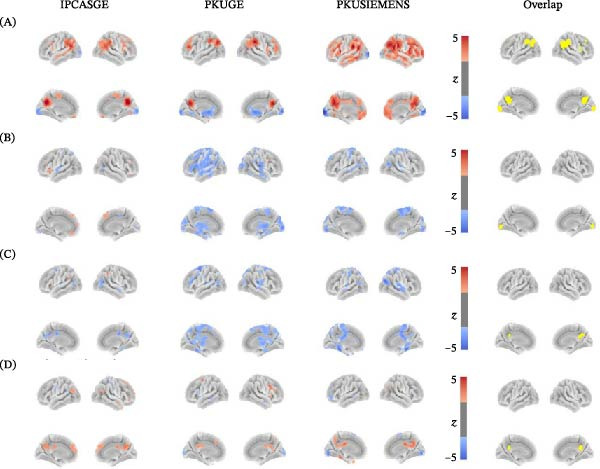
BEN differences between mental states across three MRI scanners. The first three columns show results from each scanner separately (IPCASGE, PKUGE, and PKUSIEMENS), and the last column displays the overlap of significant differences across all three scanners. (A) Task states vs. resting state. (B) Rumination vs. sad memory. (C) Distraction vs. sad memory. (D) Rumination vs. distraction. The color bar indicates *z*‐scores, with warm color indicating higher BEN and cool color indicating lower BEN.

Compared to the resting state, task states showed higher BEN in the DLPFC, temporoparietal junction (TPJ), and posterior cingulate cortex/precuneus (PCC/PCu), along with lower BEN in the visual cortex (VC) (Figure 1A). Compared to sad memory, rumination showed lower BEN in the VC, and distraction showed lower BEN in the PCC/PCu (Figure 1B,C). Compared to distraction, rumination exhibited a higher BEN in the PCC/PCu (Figure 1D).

**Figure 2:**
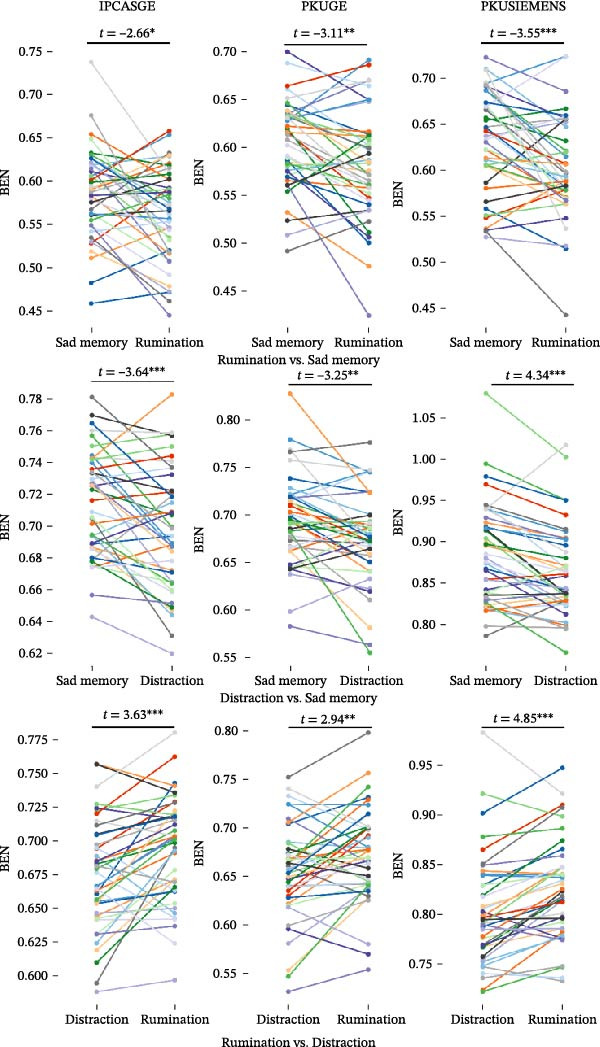
Mean BEN values across mental states in overlapping brain regions. Mean BEN values extracted from overlapping brain regions (VC or PCC/PCu, as identified in Figure 1) are shown for each participant and each condition. The *x*‐axis denotes the different task states, and the *y*‐axis represents the mean BEN values. Individual participants are distinguished by different line colors. Paired *t*‐test results (*t*‐values and significance levels) are indicated as follows:  ^∗^ *p* < 0.05,  ^∗∗^ *p* < 0.01, and  ^∗∗∗^ *p* < 0.001.

Mean BEN values were subsequently extracted from the VC and PCC/PCu for each participant during each condition. The results demonstrated significant differences across the three task states, with findings replicated across all three scanners (Figure 2): BEN during rumination was significantly lower than during sad memory (IPCASGE: *t* = −2.66, *p* = 0.011; PKUGE: *t* = −3.11, *p* = 0.003; and PKUSIEMENS: *t* = −3.55, *p* < 0.001) in the VC, and rumination exhibited higher BEN compared to distraction (IPCASGE: *t* = 3.63, *p* < 0.001; PKUGE: *t* = 2.94, *p* = 0.005; and PKUSIEMENS: *t* = 4.85, *p* < 0.001) in the PCC/PCu, while distraction showed lower BEN relative to sad memory (IPCASGE: *t* = −3.64, *p* < 0.001; PKUGE: *t* = −3.25, *p* = 0.002; and PKUSIEMENS: *t* = −4.34, *p* < 0.001) in the PCC/PCu.

## 4. Discussion

We assessed the effects of rumination, sad memory, and distraction on BEN using repeated measurements from the same cohort across three different MRI scanners. Compared to the resting state, all task states demonstrated increased BEN in transmodal association cortices [27, 53], including the DLPFC, TPJ, and PCC/PCu. In contrast, decreased BEN was observed in the unimodal VC. Lower BEN in the VC was found during rumination compared to sad memory. Importantly, we observed decreased BEN during distraction compared to sad memory and increased BEN in PCC/PCu during rumination compared to distraction. The reproducibility of these results across multiple scanners and vendors underscores the robustness of BEN as a neuroimaging biomarker for ruminative processes. Given that multiple comparison correction was not applied in the voxel‐wise analyses, the findings reported above should be interpreted as exploratory. Nonetheless, the reproducibility of these results across multiple scanners and vendors provides empirical support for the robustness of BEN as a neuroimaging measure for characterizing ruminative processes, and future confirmatory studies focusing on the identified regions are warranted.

BEN quantifies the temporal irregularity of neural activity, whereby a higher BEN corresponds to greater signal complexity and less predictable temporal dynamics [15, 22, 54–56]. Importantly, our prior work has demonstrated that regional BEN is negatively correlated with synchronization‐based FC [30, 31, 57], suggesting that BEN and synchronization capture distinct but complementary aspects of brain activity. From the perspective of normal brain function, higher temporal coherence (i.e., lower BEN) [58] may facilitate greater spatial synchronization among regions, whereas lower coherence may reduce it. These relationships between BEN, signal complexity, and synchronization are central to interpreting the findings reported below.

The PCC/PCu is a core hub of the DMN [59, 60], which is implicated in self‐referential and internally focused cognitive processes [61–63]. Increased BEN in the PCC/PCu was observed during rumination compared to distraction, consistent with our hypothesis that rumination leads to escalated temporal complexity in the DMN. No significant change in BEN was found in PCC/PCu during rumination when compared to sad memory. In contrast, lower BEN was observed in PCC/PCu during distraction relative to sad memory. These findings suggest that during rumination, individuals remain deeply engaged in repetitive processing of negative events, while during distraction, attention is redirected away from self‐referential processing, accompanied by reduced temporal complexity in this region. These results are consistent with studies demonstrating a close association between PCC/PCu activity and rumination [10–13, 64–67]. Burkhouse et al. [11] found that all participants, including those with remitted major depressive disorder and healthy controls, recruited the PCC during rumination, and increased activation in the PCC was correlated with greater self‐reported rumination and depressive symptoms in adolescents. More recently, using intracranial electroencephalography (iEEG), Chen et al. [66] observed enhanced low‐frequency power in the PCu during rumination compared to a control condition.

Within the broader DMN framework, the original dataset used in this study was designed to characterize DMN dynamics during ruminative states [13], and prior analyses demonstrated decreased FC within the DMN during rumination. Our findings complement and extend this work by showing that such network‐level alterations may arise, at least in part, from changes in local neural activity complexity within the PCC/PCu. Rather than reflecting widespread DMN dysfunction, the observed pattern suggests that the PCC/PCu serves as a key local hub whose elevated temporal complexity (higher BEN) disrupts the capacity of other DMN regions to maintain temporal synchronization with this hub, thereby contributing to reduced intra‐DMN FC. This interpretation is supported by our recent findings demonstrating a negative correlation between regional BEN and the average FC strength between that region and the rest of the brain [30, 31, 57]. It is also worth noting that in single‐session analyses (IPCASGE), increased BEN was observed in other canonical DMN nodes during rumination, such as the dorsomedial prefrontal cortex, suggesting that BEN changes during rumination may extend beyond the PCC/PCu under certain conditions. In depression, a study independently identified increased BEN in the right PCu relative to healthy controls, which was reduced following electroconvulsive therapy [43], further highlighting the clinical relevance of PCC/PCu BEN as a potential biomarker.

From the perspective of cognitive states, higher BEN in the PCC/PCu during rumination reflects more intense and temporally complex self‐referential thinking, which is less predictable and less compressible in terms of its uncertainty. This pattern is consistent with sustained and elaborate internal mentation. Conversely, lower BEN in the VC during rumination indicates more temporally coherent and ordered neural activity, reflecting reduced processing of external environmental information. This dissociation between high PCC/PCu BEN and low VC BEN is also consistent with reduced temporal synchronization between these two regions during rumination [57, 68]. During distraction, the reversed pattern (relatively lower BEN in the PCC/PCu and higher BEN in the VC) suggests reduced self‐referential processing and greater engagement with external information, with the VC exhibiting richer and more flexible temporal dynamics. Notably, all mental states showed reduced VC BEN compared to the resting state, with rumination yielding the lowest VC BEN, followed by distraction and sad memory. This gradient likely reflects a progressive shift in attentional focus away from external environmental information, with rumination imposing the greatest suppression on VC temporal complexity given its repetitive, internally focused nature. Although lower VC BEN during rumination relative to distraction was not replicated across all three scanners, it was consistently observed on two scanners (PKUGE and PKUSIEMENS) (Figure 1D) and is further supported by evidence that self‐reported rumination tendency is associated with reduced VC activation in nondepressed individuals [69]. In depression, abnormal VC functioning has been implicated in both the onset of illness and treatment response [38, 70, 71]: Lower VC activity during emotional processing predicts a better response to scopolamine [70], and our prior work found that greater BEN increase in the VC is associated with better clinical improvement after 8 weeks of treatment [38].

Several limitations must be acknowledged. First, we did not apply multiple comparison corrections to the voxel‐wise analyses. As noted above, the findings should therefore be regarded as exploratory, and future studies with larger samples and confirmatory designs are needed to establish the robustness of the implicated regions. We note, however, that the replication of key findings across three independent scanners from different vendors and sites provides a form of empirical cross‐validation that mitigates, though does not eliminate, the concern for false positives [72, 73]. Furthermore, the brain regions identified, particularly the PCC/PCu within the DMN, are well established in the rumination literature [12, 68], reducing the a priori likelihood of these being false positives. Second, although our paired *t*‐test design inherently controls for stable interindividual differences such as age and sex, we conducted additional post hoc regression analyses using within‐subject difference scores to examine potential modulating effects of these variables. The results indicated that age did not significantly modulate the key task contrasts in either the PCC/PCu or VC across any scanner. A significant sex effect was observed in the PCC/PCu contrast for the PKUSIEMENS scanner (*p* = 0.029) only, but this did not replicate across the other two scanners and should therefore be interpreted with caution. These supplementary analyses suggest that the main findings are not systematically confounded by age or sex, which is further supported by the relatively balanced sex distribution (19 males and 22 females) and homogeneous age range (18–35 years) of our sample. Third, this study utilized healthy adult participants, and the cognitive processes involved in eliciting ruminative states may differ between healthy individuals and patients with depression. Our findings therefore cannot be directly generalized to clinical populations. Additionally, research has demonstrated that rumination is influenced by sex and age [74–76], which warrants consideration in future studies with more diverse samples. Finally, the application of other entropy‐based algorithms may enhance the interpretability of the results [77, 78].

In conclusion, this exploratory study consistently demonstrated decreased BEN in the VC during rumination compared to sad memory and increased BEN in the PCC/PCu during rumination compared to distraction across multiple MRI scanners, suggesting that rumination is associated with heightened temporal complexity in regions supporting internal self‐referential processing and reduced temporal complexity in regions supporting external information processing. This neural pattern aligns with the conceptualization of rumination as involving repetitive, self‐referential thinking focused on the negative aspects of the self. The use of BEN provides a novel perspective for understanding the neural underpinnings of rumination and offers a promising biomarker framework for investigating its role in depression and related mental disorders. Future confirmatory studies with larger samples and multiple comparison corrections are needed to establish the generalizability of these findings.

## Author Contributions

**Jue Lu**: formal manuscript drafting, comprehensive reanalysis and validation of data, preparation of all figures, critical revision of the results and their interpretation. **Donghui Song**: core conceptualization and methodological design; original data analysis; supervision of analytical workflows; revision of the Section 1, Section 4, and Section 2. **Da Chang**: conceptualization and methodological design, manuscript review and critical editing. **Xiao Zhang**: substantive manuscript revision, critical review of the theoretical background and Section 4, identification and correction of logical and contextual inconsistencies. **Xiaoye Ma**: detailed manuscript review and revision, independent verification of analytical results, provision of research resources, acquisition of funding. **Ze Wang**: overall conceptualization, supervision of the entire research project, methodological guidance, final manuscript revision and oversight.

## Funding

Xiaoye Ma is supported by the Qihang Program of Shanghai Mental Health Center (Grant 2025‐QH‐04).

## Ethics Statement

The data used in this study are publicly available and were obtained from http://rfmri.org/RuminationfMRIData. According to the original study [13], the dataset was collected following appropriate ethical guidelines, and all necessary approvals were obtained by the original data collectors. However, the specific approval ID or number is not publicly disclosed by the dataset. Detailed information on the ethical oversight for the data collection can be found at [13].

## Conflicts of Interest

The authors declare no conflicts of interest.

## Data Availability

The original data are available from the RMP Rumination fMRI Dataset (http://rfmri.org/RuminationfMRIData or https://doi.org/10.57760/sciencedb.o00115.00002). The BEN maps and statistical maps can be accessed from OSF (https://osf.io/r42yv/?view_only=3d82a0a04db04983957e0542c99a531a).
